# Regulation of Human Natural Killer Cell IFN-γ Production by MicroRNA-146a *via* Targeting the NF-κB Signaling Pathway

**DOI:** 10.3389/fimmu.2018.00293

**Published:** 2018-03-09

**Authors:** Hongwei Wang, Yibo Zhang, Xiaojin Wu, Yufeng Wang, Hanwei Cui, Xinxin Li, Jianying Zhang, Norman Tun, Yong Peng, Jianhua Yu

**Affiliations:** ^1^The Ohio State University Comprehensive Cancer Center, Columbus, OH, United States; ^2^Department of Pathology, the First Affiliated Hospital, Chinese PLA General Hospital, Beijing, China; ^3^The First Affiliated Hospital of Soochow University, Suzhou, China; ^4^Department of Obstetrics and Gynecology, Daping Hospital, Army Medical School, Chongqing, China; ^5^Department of Bioinformatics, The Ohio State University, Columbus, OH, United States; ^6^State Key Laboratory of Biotherapy, West China Hospital, Sichuan University, Chengdu, China; ^7^Division of Hematology, Department of Internal Medicine, The Ohio State University, Columbus, OH, United States; ^8^The James Cancer Hospital, Columbus, OH, United States

**Keywords:** natural killer cells, microRNA, IFN-γ, miR-146a, IRAK1, TRAF6, NF-κB

## Abstract

Natural killer (NK) cells are one group of innate lymphocytes that are important for host defense against malignancy and viruses. MicroRNAs (miRNAs) play a critical role in regulating responses of immune cells including NK cells. Accumulating evidence suggests that miR-146a is involved in the regulation of immune responses. However, the mechanism by which miR-146a regulates NK cell function is largely unknown. In the current study, we found that miR-146a intrinsically regulated NK cell function. Forced overexpression of miR-146a decreased IFN-γ production, whereas downregulation of miR-146a by anti-miR-146a significantly enhanced IFN-γ production in the human NK-92 cell line and primary human NK cells upon stimulation with IL-12 or co-stimulation with IL-12 and IL-18. Mechanistically, miR-146a regulated IFN-γ production *via* NF-κB, as evidenced in NK-92 cells, by downregulation of NF-κB p65 phosphorylation when miR-146a was overexpressed but upregulation of NF-κB p65 phosphorylation when anti-miR-146a was overexpressed. miR-146a directly targeted IRAK1 and TRAF6, the upstream signaling components of the NF-κB signaling pathway. This direct targeting mechanism confirmed the above gain- and loss-of-function approaches. However, the potent IFN-γ-producing subset, CD56^bright^ NK cells, expressed higher levels of miR-146a than the lesser IFN-γ-producing subset, CD56^dim^ NK cells. We also observed that co-stimulation of IL-12 and IL-18 significantly increased miR-146a expression in bulk NK cells and in the CD56^bright^ subset in a time-dependent manner, correlating with augmented IFN-γ production. These data suggest that miR-146a plays a negative role in IFN-γ production by human NK cells and this miRNA may be critical in preventing NK cells from being super activated and overproducing IFN-γ.

## Introduction

Natural killer (NK) cells are a subset of innate lymphoid cells. NK cells have at least two functions, cytotoxicity and cytokine secretion, which are important for intracellular pathogen defense and tumor immune surveillance (1). NK cells develop from the common lymphoid progenitor and undergo a unique developmental pathway without DNA rearrangement of a clonal antigen receptor (2). NK cell functions are controlled by a balance of various positive and negative surface receptors (3). Stimulated by monokines, such as IL-12 and IL-18 or tumor cells, NK cells produce IFN-γ, a critical type II interferon cytokine (4, 5). IFN-γ is important in promoting maturation of monocytes as well as activating monocytes/macrophages and enhancing their functions. Rapid IFN-γ production plays a critical role in defending against infectious pathogens by promoting both innate and adaptive immune responses (1, 3). Deficiency in NK cell-mediated IFN-γ production is associated with an increased incidence of both malignancy and infection (6). Regulation of NK cell IFN-γ production is a complex process which involves the integration of various signaling pathways, receptors, and transcription factors that can play either positive or negative roles (7–11).

MicroRNAs (miRNAs) are non-coding RNAs that play critical roles in various biological processes, mainly through gene regulation. mRNAs repress gene expression through translational inhibition or mRNA degradation after their binding to targeting sites in the 3′ UTR of mRNAs. miRNAs have been shown to have a wide variety of roles in cancer, inflammation, and immune responses (12–15). Recent advances highlight the importance of miRNA in regulating NK cell development, maturation, and functions (16–20). For example, miR-181a/b has been reported to upregulate IFN-γ production by human NK cells activated by the co-stimulation of IL-12 and IL-18 (21). Trotta et al. reported that by directly targeting SHIP1, miR-155 functions as a positive regulator of NK cell IFN-γ production stimulated by IL-12 and IL-18 or CD16 (22). The miR-15/16 family was predicted through bioinformatics algorithms to target the murine IFN-γ 3′ UTR, which was confirmed *in vitro* by luciferase assays (23). Furthermore, mature miRNAs from this family are downregulated in primary murine NK cells upon activation, suggesting that the miR-15/16 family plays a role in regulating NK cell IFN-γ production (23).

The miR-146 family consists of two evolutionarily conserved miRNA genes, miR-146a and miR-146b, which are located on chromosomes 5 and 10, respectively (13). miR-146a is strongly induced after challenging cells with bacterial endotoxin and may act as a fine-tuning regulator to prevent an overstimulation during inflammatory responses (24). Accumulating evidence suggests that miR-146a is involved in the regulation of the adaptive as well as the innate immune response, tumor progression, and virus infection (25). Nevertheless, more research remains to be conducted to fully understand its role and mechanism in regulating NK cell function, which may provide additional basis for a potential therapeutic role of miR-146a.

In this study, we examined the expression of miR-146a in human NK cells and its role in the regulation of IFN-γ expression, using multiple approaches, including gain- and loss-of-function studies. Our data demonstrate that miR-146a negatively regulates IFN-γ production in NK cells by targeting IRAK1 and TRAF6, with subsequent inhibition of the NF-κB signaling cascade. miR-146a likely plays a critical role in restricting IFN-γ production in super activated NK cells, as co-stimulation of IL-12 and IL-18 upregulates miR-146a and it has a higher expression level in CD56^bright^ NK cells compared to CD56^dim^ NK cells.

## Materials and Methods

### NK Cell Preparations

Primary human NK cells were freshly isolated from leukopaks of healthy individuals (American Red Cross, Columbus, Ohio, USA), using MACSxpress^®^ NK cell isolation kit (Miltenyi Biotec). The manufacturer’s protocol was followed with some modifications. An erythrocyte depletion kit (Miltenyi Biotec) was used to remove erythrocytes if cell pellets contained a significant fraction of erythrocytes. The purity of the isolated CD56^+^CD3^−^ NK cells was usually over 97%, assessed by flow cytometric analysis after staining with CD56-allophycocyanin (APC) (Beckman Coulter) and CD3-fluorescein isothiocyanate (FITC) Abs (BD Biosciences). CD56^bright^ and CD56^dim^ NK cell subsets were sorted by a FACS Aria II cell sorter (BD Biosciences) based on CD56 cell surface density after staining with CD56-APC and CD3-FITC Abs. The purity of CD56^bright^ and CD56^dim^ subsets was >98%. All work with human materials was approved by the institutional review board of The Ohio State University.

### Lentiviral Infection of Primary Human NK Cells and the NK-92 Cell Line

Lentiviral vectors encoding miR-146a (lenti-miR-146a), anti-miR-146a (miRZip-146a), and corresponding empty vectors (miR-vector and anti-miR-vector) were obtained from SBI System Biosciences. NK-92 cells and primary NK cells were infected following a protocol similar to what has been previously published (26, 27). Briefly, 293T cells were seeded onto a 15-cm dish in Dulbecco modified Eagle medium (Invitrogen) containing 10% FBS and grown for 16–18 h to 80% confluence before transfection by calcium phosphate-DNA precipitation (ProFection^®^ Mammalian Transfection System, Promega). A lentiviral construct or its corresponding empty vector (200 µg) and the packaging plasmids, VSVG (100 µg) and deltaR9 (150 µg), were used to prepare DNA precipitates. Viral supernatants from 293T cells transfected with miR-vector, miR-146a, anti-miR-vector, or anti-miR-146a were harvested at 48 h, followed by centrifugations to remove cells and cell debris. To infect purified CD56^+^ primary human NK cells, the cells were cultured at 0.8–1.0 × 10^6^ cells per well in multiple wells of a 96-well plate (round bottom) with RPMI-1640 (Invitrogen) containing 20% FBS, 900 U/ml rhIL-2, and 16 µg/ml polybrene. The lentivirus was then added. The cells were mixed well with viruses and cultured for 2 h in a 37°C incubator supplied with 5% CO2, followed by spinning in a standard swinging bucket table-top centrifuge at 400 *g* for 1 h at 30°C. This infection procedure of 2 h culture plus 1 h spin was repeated three times. Infected primary human NK cells were cultured for 3 days post infection and then sorted for GFP^+^ cells. For the infection of NK-92 cells, cells were cultured at 0.2–0.3 × 10^6^ cells per well in a 96-well plate with RPMI-1640 containing 20% FBS, 900 U/ml rhIL-2, and 16 µg/ml polybrene. Lentivirus was then added and cultured overnight at 37°C in 5% CO2. After infection, primary NK cells and NK-92 cells were sorted for GFP expression on a FACSAria II (BD Bioscience). GFP^+^CD56^+^ primary NK cells were used for experiments immediately after sorting, while GFP^+^ NK-92 NK cells were expanded before being used. The expression of miR-146a in the sorted GFP positive cells was confirmed by qRT-PCR.

### Cell Culture Conditions

The human NK cell line NK-92 was maintained in RPMI-1640 medium (Invitrogen), supplemented with 50 mg/ml penicillin, 50 mg/ml streptomycin, 20% heat-inactivated FBS (Invitrogen), and 150 U/mL rhIL-2 (Hoffman-LaRoche). Before cytokine stimulation, NK-92 cells were starved for IL-2 for ~24 h. NK-92 and purified primary NK cells were next incubated in RPMI-1640 media plus 20% FBS at 37°C with or without the addition of rhIL-12 (10 ng/mL) and/or rhIL-18 (100 ng/mL) (R&D Systems) for 24 h or the indicated time.

### Quantitative (q) RT-PCR

Total RNA from NK-92 cells or primary NK cells was extracted using miRNeasy Mini Kit (Qiagen). cDNA was generated with random hexamers according to the manufacturers’ instructions (Invitrogen) or RT primers specific for miR-146a and *RNU44* as control (Applied Biosystems) using TaqMan^®^ MicroRNA Reverse Transcription kit. Real-time reverse transcription (RT)-PCRs were performed with primer/probe sets specific for the human miR-146a, RNU44 (Applied Biosystem), *IFNG, RelA (p65), IRAK1, TRAF6*, and *18S rRNA* using an ABI prism 7700 sequence detector (Taqman; PE Applied Biosystems). Data were analyzed according to the comparative CT method and normalized to the internal control RNU44 (for miR-146a) or *18S rRNA* (for non-miRNA genes). Results (mean ± SD of triplicate wells) are represented as fold changes of expression levels.

### Immunoblotting Analysis

Cells were harvested, washed once with ice-cold PBS, and lysed with RIPA buffer [150 mM NaCl, 1% NP-40, 50 mM Tris–HCl, pH 7.5, 0.1% SDS, and 0.5% sodium deoxycholate supplemented with 1 × protease inhibitor cocktail (Roche Applied Science)]. Protein concentrations were determined by a bicinchoninic acid protein assay kit (Novagen). Protein samples were run on a 12% SDS-PAGE gel and transferred onto a PVDF membrane (Millipore) at 100 V for 2 h. Membranes were blocked in 5% skimmed milk and then incubated with primary antibody at 4°C overnight. Membranes were washed three times for 15 min each with Tris-buffered saline, 0.1% Tween 20 buffer. A horseradish peroxidase-conjugated secondary antibody was incubated with the membrane for 1 h and followed by three washes as mentioned above. The membranes were developed by the SuperSignal West Femto developing reagent from Pierce Biotechnology. Monoclonal or polyclonal Abs used were: goat monoclonal anti-β-actin from Santa Cruz Biotechnology; rabbit polyclonal anti-IRAK1, rabbit monoclonal anti-TRAF6, rabbit monoclonal anti-NF-κB p65, and rabbit monoclonal anti-phospho-NF-κB p65 from Cell Signaling Technology.

### Enzyme-Linked Immunosorbent Assay (ELISA) for IFN-γ

After the treatment described above, cell-free supernatants were collected and frozen at −80°C, thawed only once, and analyzed by ELISA according to the manufacturer’s instructions as previously described (11). Briefly, IFN-γ production was measured by a standard sandwich cytokine ELISA procedure to assess the cytokine quantity. Standards (recombinant cytokines at 156.25, 312.5, 625, 1500, 3,000, and 6,000 pg/ml concentrations) and samples were added in each well. Cytokine quantities in the samples were calculated from standard curves of recombinant cytokines using a linear regression model. Absorbance results were assessed using a microplate reader at wavelengths 450 nm for sample reading and 570 nm for background measurement. Results are shown as the mean of triplicate wells ± SD.

### Flow Cytometric Analysis

Cells were labeled with antibodies for 15 min in the dark at room temperature and then analyzed using LSRII Flow Cytometer (BD Bioscience). The anti-NF-κB antibody was purchased from Cell Signaling Technology and the anti-p-NF-κB antibody was purchased from BD Bioscience.

### Luciferase Reporter Assay

HeLa or 293T cells were seeded into 24-well plates at a concentration of 10^5^ per well and transfected 18 h later using Lipofectamine 2000 (Invitrogen) according to the manufacturer’s protocol. Each transfection reaction contained 0.6 µg of a firefly luciferase reporter vector or a mutant vector, 2.4 µg of a miR-146a or an empty vector, and 0.02 µg of a pRL-TK control vector (Promega). Cells were harvested after 48 h of transfection and assessed for luciferase activity by a Dual Luciferase Reporter Assay System (Promega). Luciferase reporter vectors or mutant vectors used were: IRAK1-UTR (Addgene), IRAK1mut-UTR (Addgene), TRAF6-UTR (Addgene), and TRAF6mut-UTR (Addgene). All assays were performed in triplicate and all values were normalized for transfection efficiency against renilla luciferase expression derived from the co-transfected pRL-TK plasmid. The means ± SD of the triplicate values are reported.

### Statistical Analysis

Graphical analysis and statistics were performed with GraphPad Prism 5.0. Student’s two-tailed *t*-test and one-way ANOVA were used as appropriate. The *p* values were adjusted for multiple comparisons using the Bonferroni method. A *p*–value <0.05 was considered statistically significant.

## Results

### The Negative Role of miR-146a in Regulating IFN-γ Production by IL-12 and/or IL-18-Stimulated Human NK Cells

**Figure 1:**
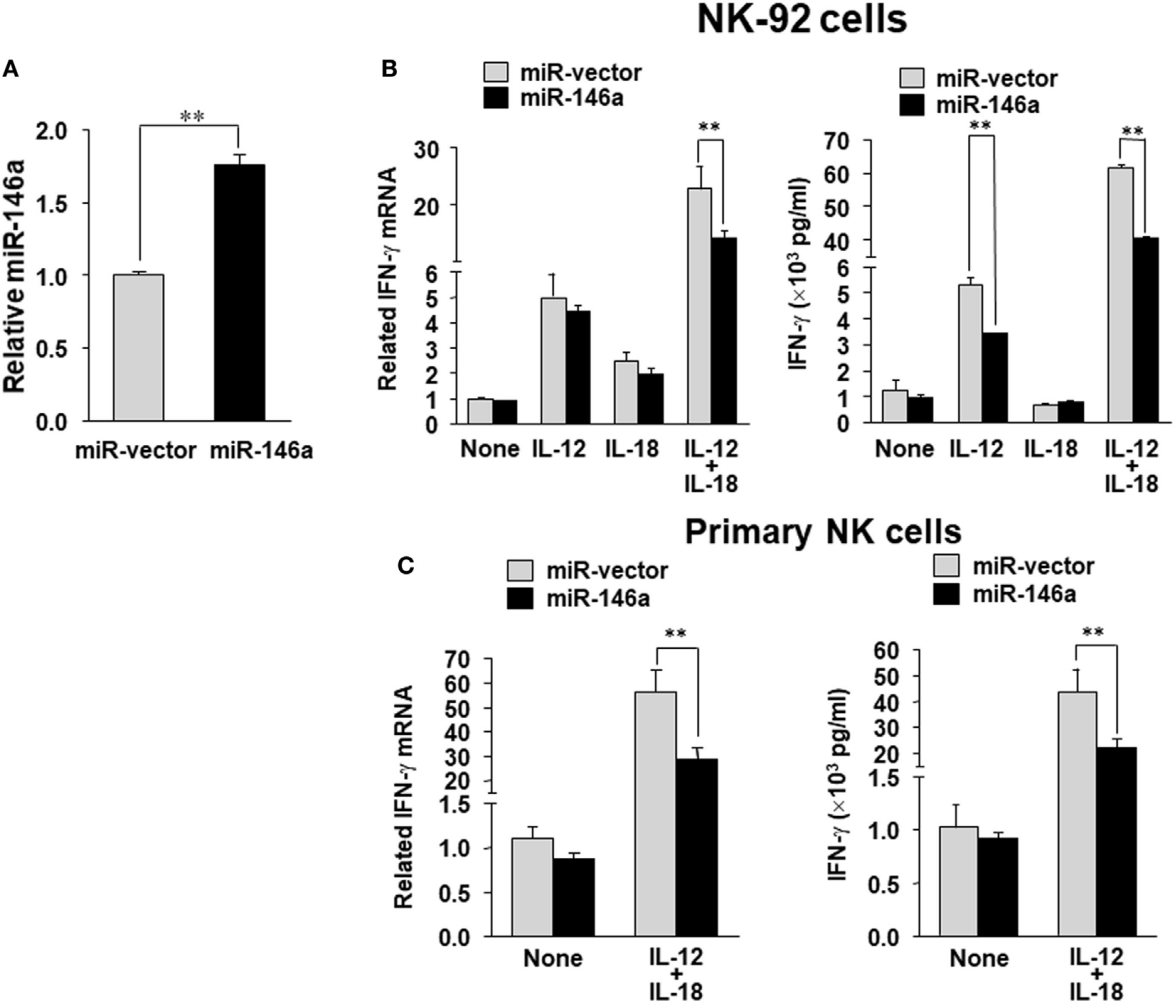
miR-146a overexpression decreases IL-12 plus IL-18–induced IFN-γ production in NK cells. NK-92 cells were infected with the lentivirus containing GFP (miR-vector) or GFP and miR-146a. Cells were then sorted for high GFP expression and analyzed for miR-146a expression **(A)**. NK-92 cells **(B)** or primary human CD56^+^ NK cells **(C)** were infected using either miR-vector or miR-146a, sorted by FACS for GFP, and co-stimulated with IL-12 (10 ng/mL) plus IL-18 (100 ng/mL) for 24 h. Cell pellets were collected for RNA preparation and cDNA synthesis, followed by assessment of IFN-γ gene expression by quantitative (q)RT-PCR. Supernatants were collected to measure IFN-γ protein levels by enzyme-linked immunosorbent assay (ELISA). The data shown are representative of at least four experiments with similar results. Data are reported as mean ± SD. *p < 0.05; **p < 0.01. Error bars represent SD.

To evaluate the impact of miR-146a on IFN-γ production in NK cells, we overexpressed miR-146a in the human NK cell line NK-92 using a lentiviral vector carrying either the cDNA for GFP alone (miR-vector) or GFP and the miR-146a native stem–loop structure along with 200 to 400 base pairs of upstream and downstream flanking genomic sequence (miR-146a). Overexpression of miR-146a was confirmed by qRT-PCR in the GFP^+^ NK-92 cells (Figure 1A). When miR-146a was overexpressed, we observed a significant decrease of IFN-γ expression in NK-92 cells at both the mRNA and the protein levels after stimulation with IL-12 plus IL-18, compared to NK-92 cells infected with empty vector control. In the case of single cytokine stimulation, i.e., IL-12 or IL-18 alone, the inhibition of IFN-γ by miR-146a was only observed at the protein level when NK-92 cells were treated with IL-12 (Figure 1B). We then repeated the experiment in primary human NK cells stimulated by IL-12 plus IL-18. Similar to what we observed in NK-92 cells, overexpression of miR-146a in primary human NK cells led to a significant suppression of IFN-γ expression upon the co-stimulation of IL-12 and IL-18 at both the mRNA and protein levels (Figure 1C).

**Figure 2:**
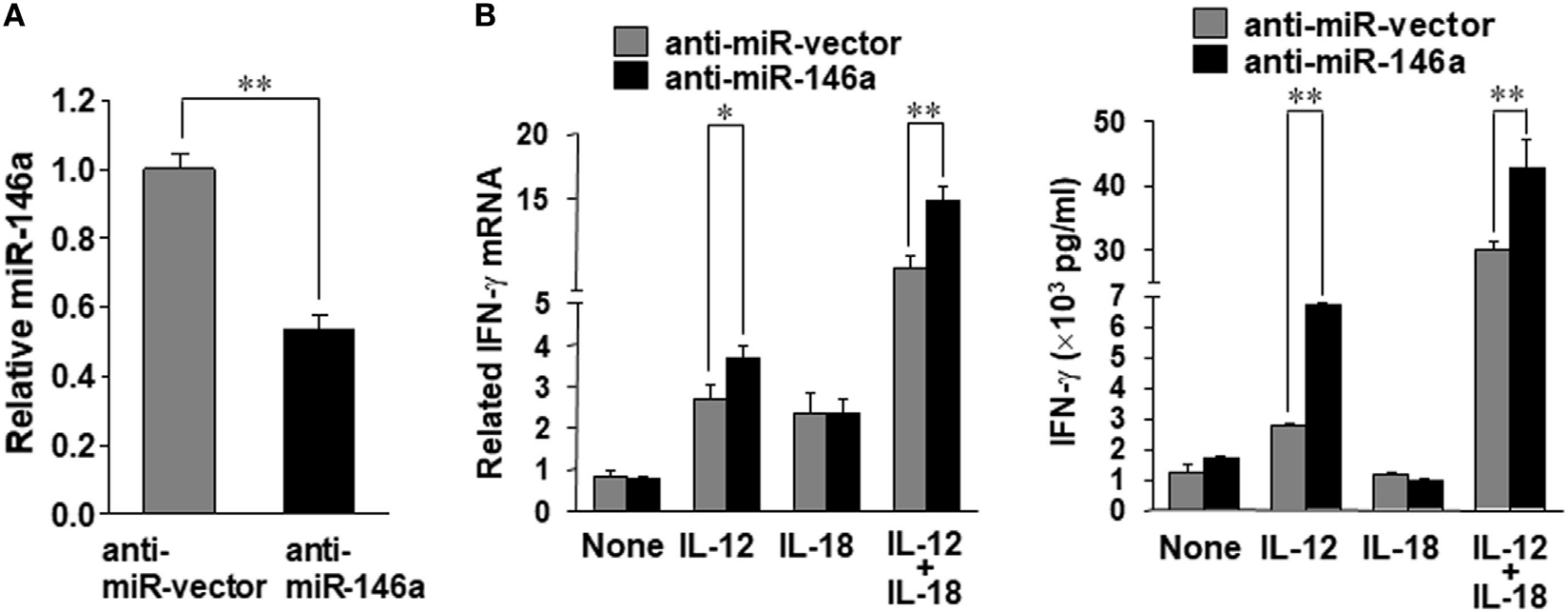
miR-146a knockdown enhances IL-12 plus IL-18–induced IFN-γ production in NK cells. NK-92 cells were infected with an empty vector (anti-miR-vector) or a miR-146a antisense encoding vector (anti-miR-146a), sorted for GFP^+^ and analyzed for miR-146a expression **(A)**. The NK-92 stable cell line with miR-146 overexpression or an empty vector was co-stimulated with IL-12 (10 ng/mL) plus IL-18 (100 ng/mL) for 24 h. Cell pellets were collected for RNA isolation and cDNA synthesis, followed by assessment of IFN-γ mRNA expression quantified by qRT-PCR. Supernatants were collected to measure IFN-γ by enzyme-linked immunosorbent assay (ELISA) **(B)**. This experiment is representative of at least four such experiments performed with similar results. Data shown are mean ± SD. *p < 0.05; **, *p* < 0.01. Error bars represent SD.

In order to confirm the data derived from overexpression experiments, we further down-modulated miR-146a by infecting NK-92 cells with the lentivirus encoding miR-146a antisense (miR-Zip146a). NK-92 cells infected with the empty lentivirus called miR-Zip00 (anti-miR-vector) served as control and GFP was used as a selection marker for FACS cell sorting. After confirming knockdown of miR-146a by qRT-PCR, the cells were treated with IL-12 or IL-18 or both, followed by the assessment of IFN-γ at both the mRNA and protein levels (Figure 2A). We observed that at both the mRNA and the protein levels, down-modulation of miR-146a significantly enhanced IFN-γ production in NK-92 cells stimulated by IL-12 alone or IL-12 plus IL-18 (Figure 2B).

Taken together, the above data implied that miR-146a played a negative role in the regulation of IFN-γ production in IL-12 plus IL-18-stimulated human NK cells.

### miR-146a Negatively Regulates NF-κB Expression and Activity in Human NK Cells

**Figure 3:**
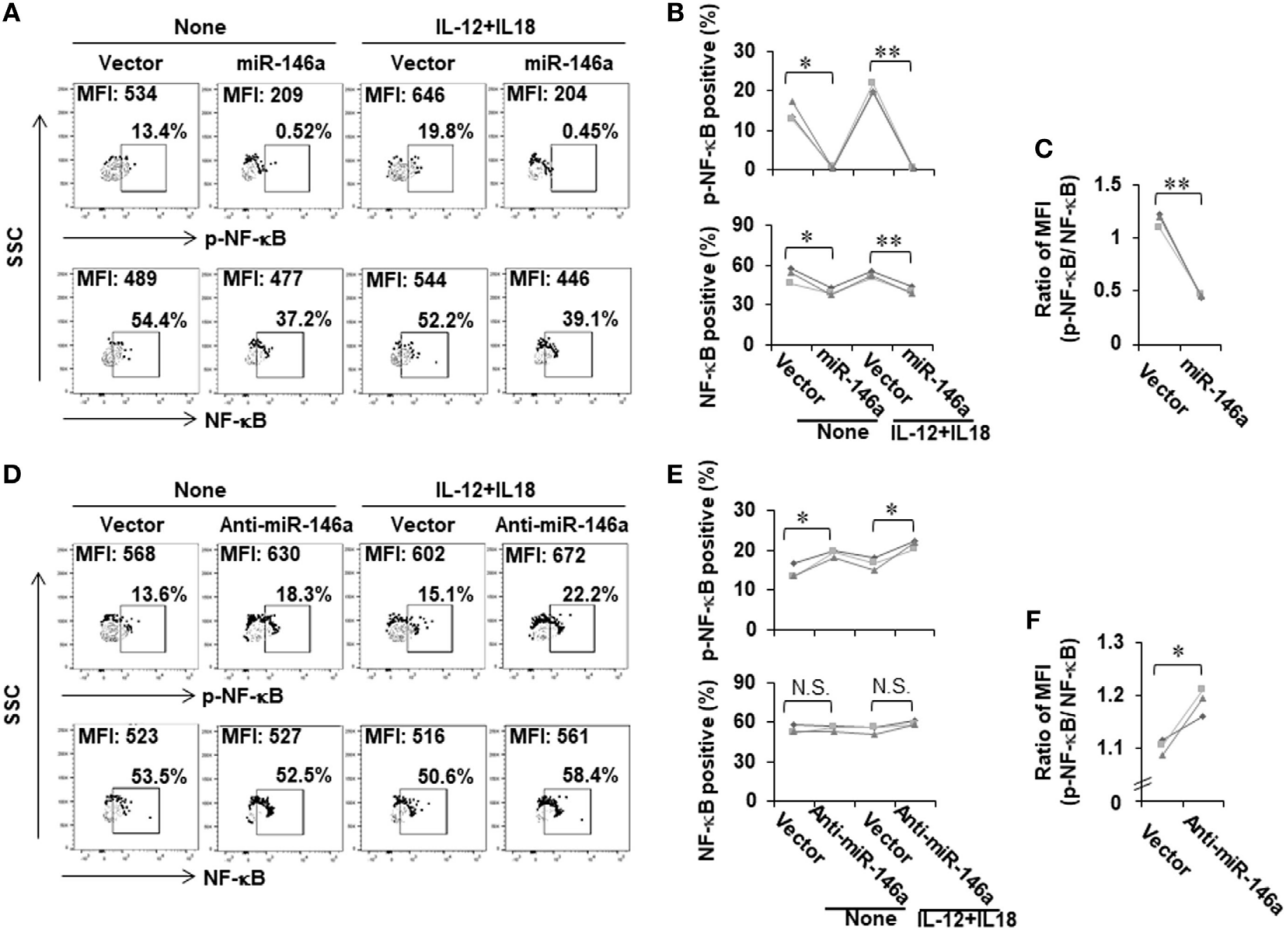
NF-κB signaling is negatively regulated by miR-146a in resting and cytokine-stimulated human NK cells. NK-92 cells expressing miR-146a or anti-mIR-146a were cultured in medium without IL-2 for 24 h, followed by treatment with or without IL-12 (10 ng/mL) plus IL-18 (100 ng/mL) for an additional 24 h. Cells were harvested to stain with antibodies, followed by flow cytometric analysis. The NF-κB p65 protein levels and its phosphorylation were measured by flow cytometric analysis **(A,B,D,E)** (*n* = 3). The ratio of p-NF-κB to NF-κB was calculated based on mean fluorescence intensity (MFI) of each molecule **(C,F)** (*n* = 3). *p < 0.05; **p < 0.01. Error bars represent SD.

We next investigated the mechanism by which miR-146a regulates IFN-γ production in NK cells. Bioinformatic analysis indicated that miR-146a did not directly target the IFN-γ gene, *IFNG*. We thus explored an indirect regulation mechanism. Previously, it has been shown that NF-κB is a positive regulator of IFN-γ production (28). We then hypothesized that miR-146a downregulates IFN-γ by targeting NF-κB signaling. To test this, we analyzed NF-κB expression and activity in NK-92 cells overexpressing sense or antisense miR-146a. Compared to empty vector-transduced NK-92 cells, NF-κB p65 was downregulated in NK-92 cells overexpressing miR-146a at the phosphorylated protein levels (Figures 3A,B; Figure S1A in Supplementary Material). The total protein levels of NF-κB p65 were also shown to be downregulated by overexpression of miR-146a at both the protein level (Figures 3A,B; Figure S1A in Supplementary Material) and the mRNA level (Figure S1 in Supplementary Material). When the ratio of phospho (p)-NF-κB to NF-κB was compared, the downregulation of NF-κB signaling by overexpression of miR-146a was more dramatic (Figure 3C). In addition, the corresponding upregulation of p-NF-κB p65 was observed in cells overexpressing antisense miR-146a (Figures 3D,E; Figure S1B in Supplementary Material), although overexpression of antisense miR-146a did not result in significant upregulation of total NF-κB (Figures 3D,E; Figure S1B in Supplementary Material). However, when the ratio of p-NF-κB to NF-κB was compared, the upregulation of NF-κB signaling by miR-146a downregulation was still significant (Figure 3F). These results regarding the regulation of p-NF-κB and NF-κB by miR-146a was also observed under the condition of the co-stimulation by IL-12 and IL-18 (Figures 3A,B,D,E). Our data suggested that miR-146a negatively regulates IFN-γ in IL-12 plus IL-18-stimulated and resting NK cells, at least partially through inhibiting the NF-κB signaling pathway.

### miR-146a Downregulates TRAF6 and IRAK1 Expression in Human NK Cells

**Figure 4:**
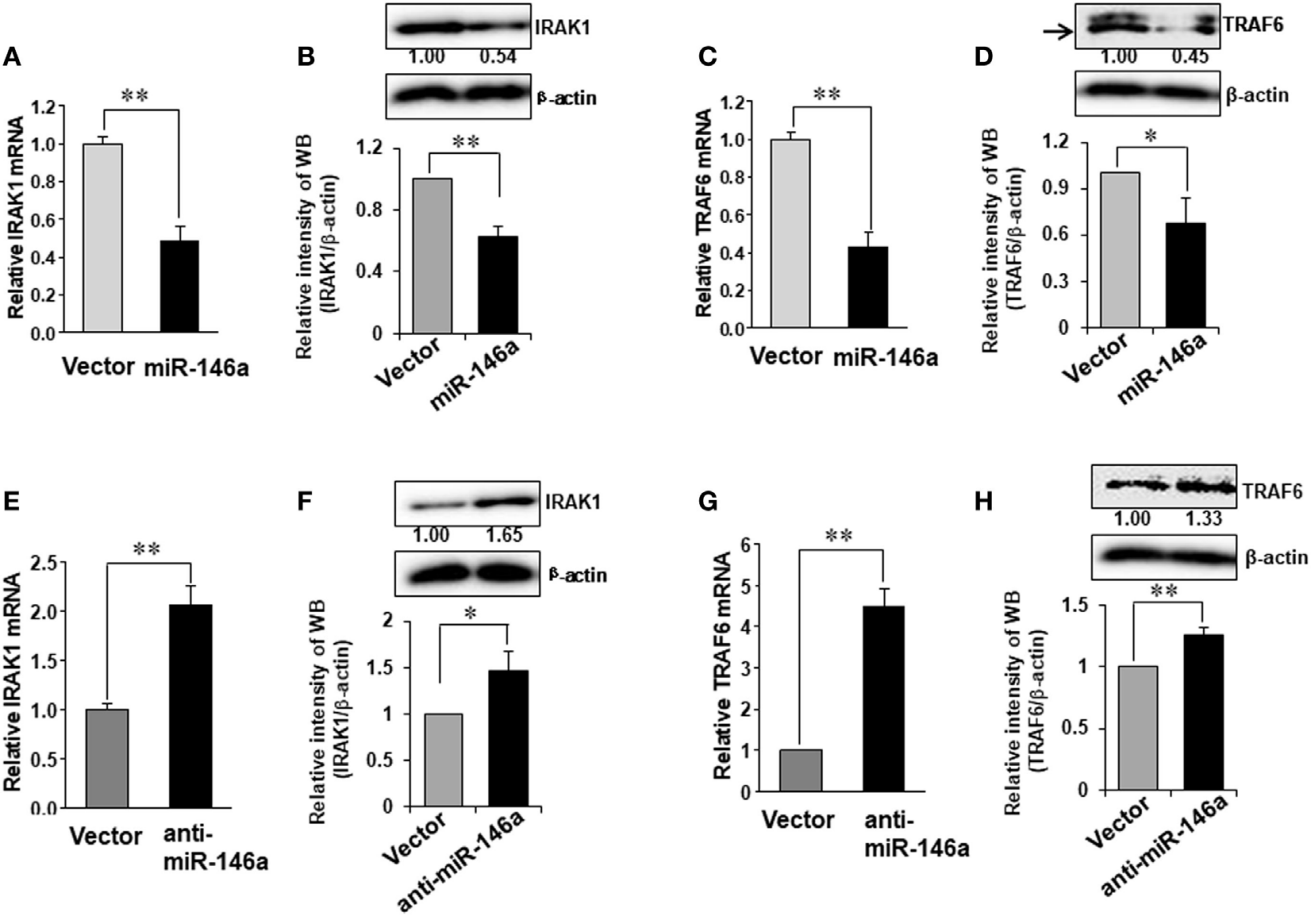
Expression of TRAF6 and IRAK1 in NK-92 cells transduced with miR-146a or anti-miR-146a. NK-92 cells expressing miR-146a or anti-miR-146a were cultured in medium without IL-2 for 24 h. Cells were harvested to extract RNA, followed by cDNA synthesis. The expression of IRAK1 **(A,B,E,F)** and TRAF6 **(C,D,G,H)** at the mRNA level and the protein level were assessed by qRT-PCR and immunoblotting, respectively (*n* = 3). Data shown are mean ± SD. *p < 0.05, **p < 0.01. Error bars represent SD.

It is reported that TRAF6 and IRAK1 are major signal transducers in the NF-κB pathway (29) and both TRAF6 and IRAK1 are suggested to be potential target genes of miR-146a in human synovial fibroblasts cells (30). However, this has not been studied in NK cells. We first investigated whether miR-146a regulated TRAF6 and IRAK1 in human NK cells. Our data demonstrated that miR-146a overexpression reduced the mRNA and the protein levels of IRAK1 and TRAF6 (Figures 4A–D). By contrast, down-modulation of miR-146a resulted in increased IRAK1 and TRAF6 at both the protein and mRNA levels, compared with the empty vector-transduced NK-92 cells (Figures 4E–H). These data suggested that miR-146a may target TRAF6 and IRAK1 to repress their expression in human NK cells.

### TRAF6 and IRAK1 Are *Bona Fide* Targets of miR-146a

**Figure 5:**
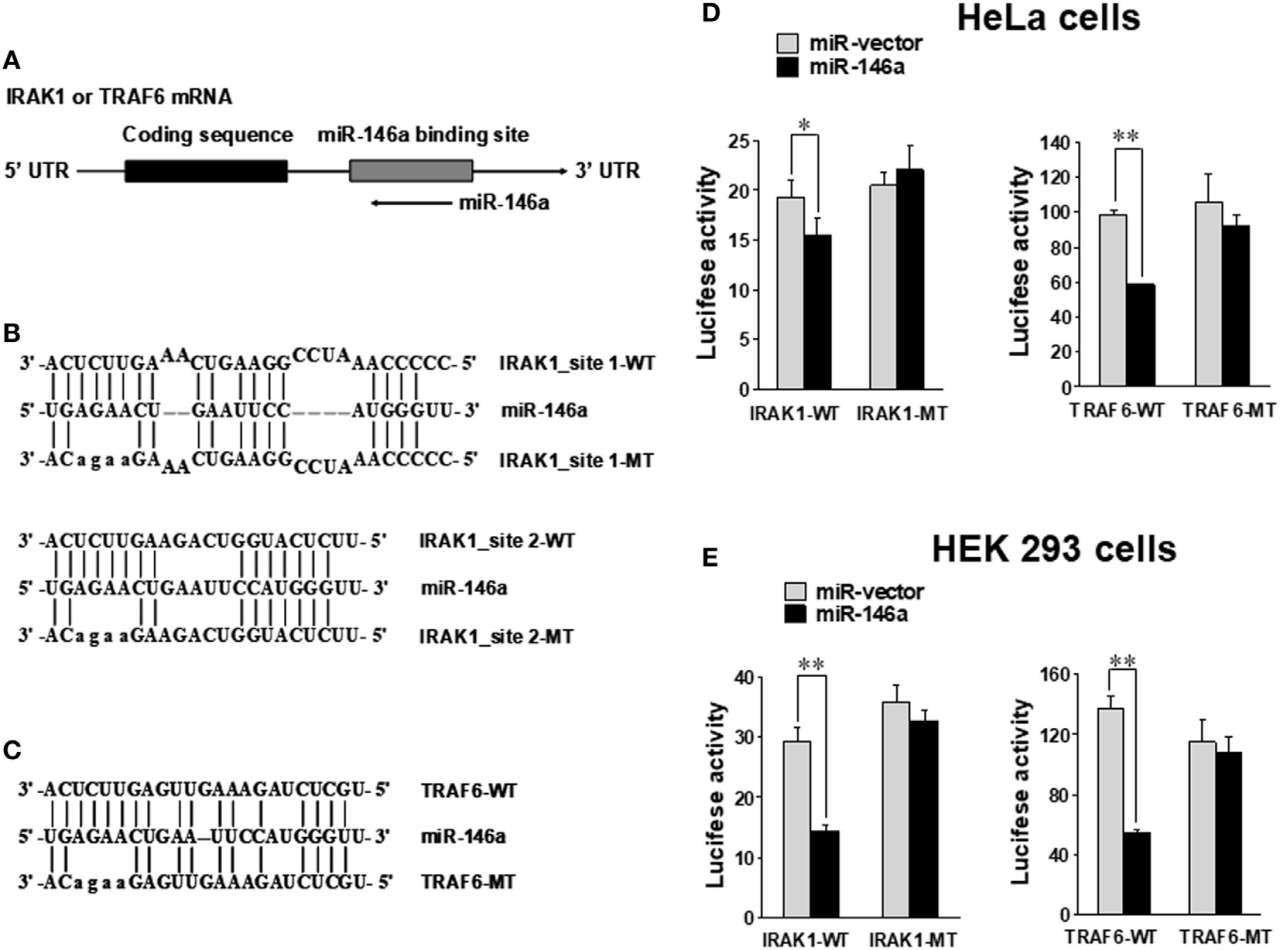
IRAK1 and TRAF6 are direct targets of miR-146a. Schematic representation of the IRAK1 and TRAF6 3′ UTR indicating the putative binding sites of miR-146a **(A)**. Sequence alignment of miR-146a and its target sites in 3′ UTRs of TRAF6 **(B)** and IRAK1 **(C)**. WT and MT indicate wild-type and mutant sequences, respectively. In the IRAK1-UTR-MT plasmid, both miR-146-binding sites were mutated as shown. HeLa cells **(D)** and 293T cells **(E)** were transiently co-transfected with either pGL3 luciferase vector containing a fragment of IRAK1 and TRAF6 3′ UTR harboring miR-146a binding sites or the corresponding mutant constructs. Luciferase activities were normalized to the activity of renilla luciferase. Data shown are representative of at least three experiments with similar results and are presented as mean ± SD. *p < 0.05; **p < 0.01. Error bars represent SD.

Using miRNA target prediction software (31), we found that there was a putative miR-146a binding site(s) in the 3′ UTR of IRAK1 or TRAF6 mRNA (Figure 5A). To test if miR-146a could directly target IRAK1 and TRAF6 mRNAs, we undertook luciferase reporter assays using plasmids harboring the wild-type 3′ UTR and a mutated 3′ UTR (Figures 5B,C) (24). HeLa and 293T cells were transfected with either the pGL3 luciferase vector containing a fragment of IRAK1 or TRAF6 3′ UTR harboring a miR-146a binding site(s) or the corresponding mutant constructs. Our data showed that the luciferase activity of the wild-type 3′ UTR of IRAK1 and TRAF6 was significantly inhibited by miR-146a in HeLa and 293T cells. By contrast, little change in luciferase activity was observed when transfected with the mutant 3′ UTR of IRAK1 or TRAF6 (Figures 5D,E). These data suggest that IRAK1 and TRAF6 are direct targets for miR-146a.

### miR-146a Expression Is Upregulated in Cytokine-Activated Primary Human NK Cells

**Figure 6:**
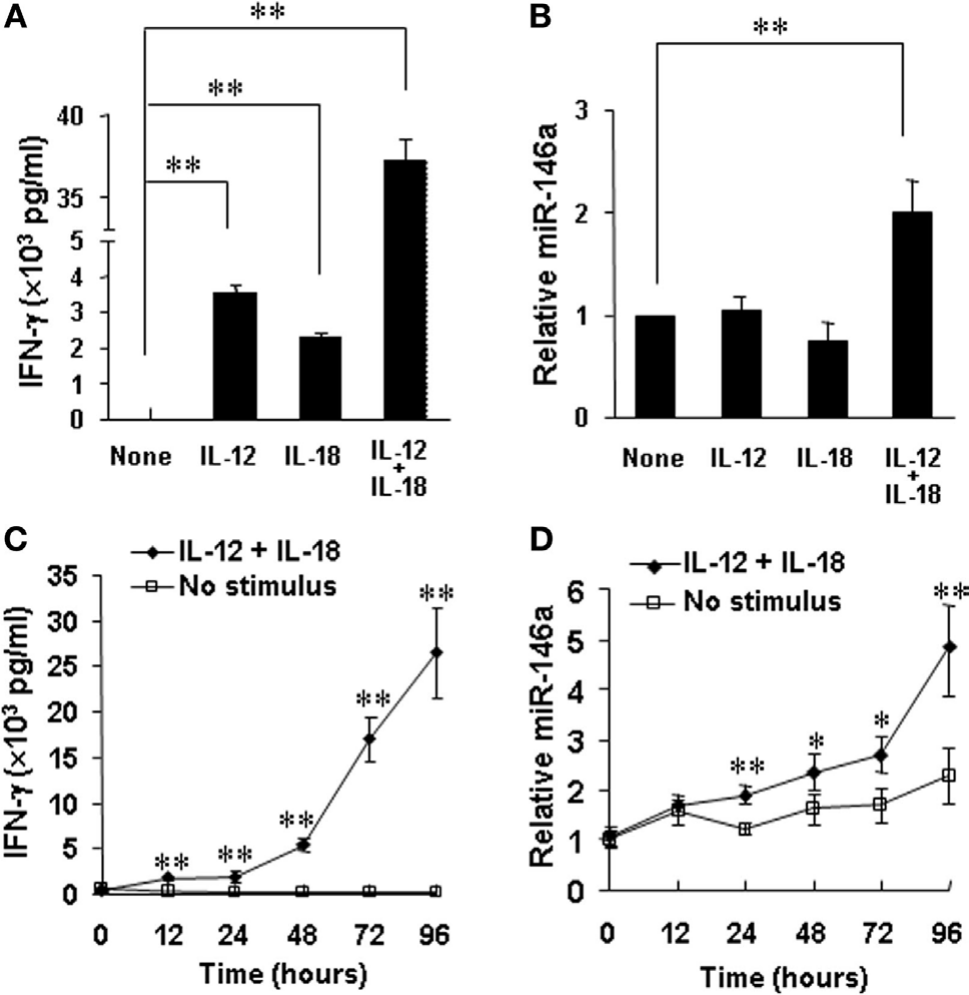
miR-146a expression in resting and IL-12 and/or IL-18-stimulated natural killer (NK) cells. Human NK cells were unstimulated or stimulated with IL-12 (10 ng/mL), IL-18 (100 ng/mL), or the combination of both for 24 h. Supernatants were harvested to be analyzed for IFN-γ production by enzyme-linked immunosorbent assay (ELISA) **(A)**. Cell pellets were collected for RNA preparation and cDNA synthesis, followed by assessment of miR-146a expression by qRT-PCR **(B)**. Human NK cells were unstimulated or co-stimulated with the combination of IL-12 (10 ng/ml) and IL-18 (100 ng/ml) for the indicated times. Supernatants were harvested to be analyzed for IFN-γ production by ELISA **(C)**. Cell pellets were collected and used to prepare RNA and cDNA synthesis, followed by assessment of miR-146a expression by qRT-PCR **(D)**. Data reported are representative of at least four experiments with similar results and are shown as mean ± SD. *p < 0.05; **p < 0.01. Error bars represent SD.

Accumulating evidence from our group and others indicates that positive regulators of IFN-γ production usually correspond to upregulation of these factors by cytokines or their combinations (e.g., IL-12 plus IL-18), while negative regulators are usually inhibited by the cytokine stimulations (10, 11, 22, 32). Our data showed that miR-146a inhibited IFN-γ production stimulated by IL-12 and IL-18 in human NK cells. We thus tested whether IL-12 and IL-18 downregulates miR-146a. For this, we stimulated human NK cells by cytokines for 24 h. As previously reported (22), stimulation with IL-12, IL-18, or their combination induced a rapid and high induction of IFN-γ expression in CD56^+^ human NK cells, especially after stimulation by IL-12 plus IL-18 (Figure 6A). However, surprisingly, expression of miR-146a was found to be markedly upregulated in NK cells stimulated with the combination of IL-12 and IL-18, compared to resting and IL-12 or IL-18-stimulated NK cells (Figure 6B). Furthermore, we stimulated CD56^+^ human NK cells by IL-12 plus IL-18 for various periods to address whether there is a time-dependent correlation between IFN-γ production and miR-146a expression in NK cells. We indeed observed that stimulation of primary human NK cells with IL-12 plus IL-18 significantly induced IFN-γ production, accompanied with significantly upregulated miR-146a expression of NK cells in a time-dependent manner (Figures 6C,D). Collectively, our data indicated that the combination of IL-12 and IL-18, one of the most potent IFN-γ stimuli, rather than less potent single cytokine can induce miR-146a expression, correlating with increased IFN-γ secretion in primary human NK cells. We then hypothesized that miR-146a plays a restrictive role in NK cells with a high IFN-γ production potential. We continued to test this hypothesis below.

### The Increase of miR-146a in the IFN-γ Secreting NK Cell Subset Correlates with NF-κB Activation

**Figure 7:**
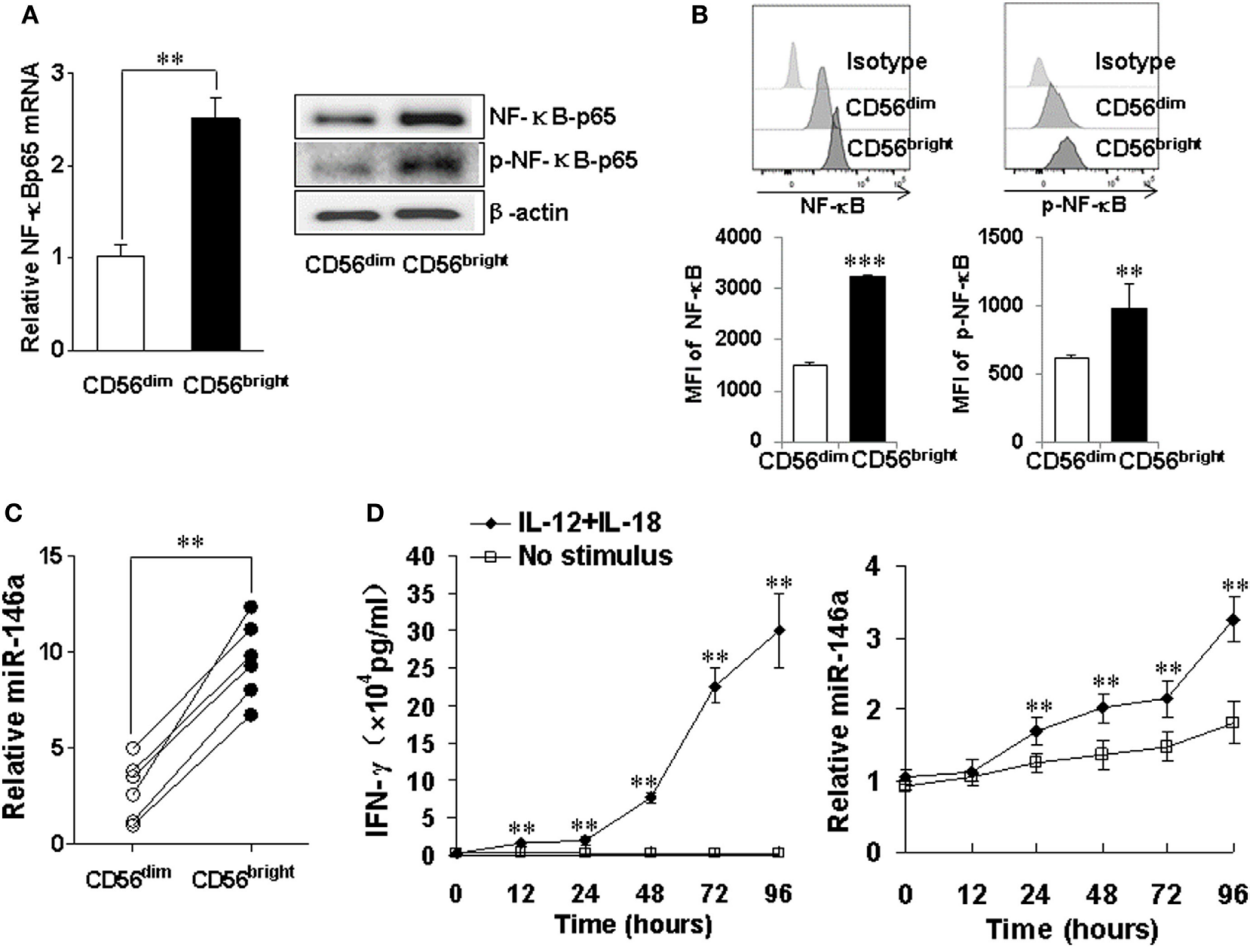
miR-146a and NF-κB expressions in natural killer (NK) cell subsets CD56^bright^ and CD56^dim^ NK cells were sorted by a FACS Aria II cell sorter based on CD56 cell surface density, followed by assessment of NF-κB p65 mRNA and protein levels by qRT-PCR and immunoblotting, respectively **(A)**. CD56^bright^ and CD56^dim^ NK cells were gated based on CD56 cell surface density and NF-κB p65 protein levels and phosphorylation levels were assessed by flow cytometric analysis **(B)**. miR-146a expression was quantified by qRT-PCR in NK cell subsets **(C)**. CD56^bright^ NK cells were left unstimulated or co-stimulated with a combination of IL-12 (10 ng/ml) and IL-18 (100 ng/ml) for the indicated times, after which the supernatants were analyzed for IFN-γ production by enzyme-linked immunosorbent assay. Cell pellets were collected and used to prepare RNA and miR-146a expression and were quantified by qRT-PCR **(D)**. This experiment is representative of at least four such experiments performed with similar results. Data shown are mean ± SD. *p < 0.05; **p < 0.01. Error bars represent SD.

CD56^bright^ and CD56^dim^ are two human NK subsets. The former has a much better capacity to produce IFN-γ upon cytokine activation (33). We analyzed NF-κB signaling in these two subsets and found higher mRNA levels as well as total and phosphorylated protein levels of NF-κB p65 in the CD56^bright^ subset (Figures 7A,B). Consistent with our data above, we also observed that the expression of miR-146a was significantly higher in the resting CD56^bright^ subset than that in the resting CD56^dim^ subset (Figure 7C). When the IFN-γ-producing CD56^bright^ subset was co-stimulated with IL-12 and IL-18, we also observed that time-dependent increases of IFN-γ correlated with corresponding increases of miR-146a (Figure 7D).

## Discussion

Natural killer cells are a critical component of innate immunity with the capacity to destroy cancer or cancer-initiating cells and clear viral infections. IFN-γ is a critical cytokine produced by activated NK cells. The critical role of IFN-γ during inflammation and tumor immunity has been well acknowledged. Overproduction of IFN-γ can result in autoimmune disorders, while deficiency results in increased susceptibility to infection and/or malignancy, suggesting that this cytokine needs to be tightly regulated (34–36). Hence, understanding molecular pathways that tightly regulate NK cell IFN-γ expression is of fundamental importance, which may lead to the discovery of potential therapeutic targets for chronic inflammation and/or cancer.

In this study, we assessed the expression of miR-146a in human NK cells and its role in the regulation of NK cell IFN-γ expression. Our current work supports a negative and restrictive role of miR-146a in regulating NK cell IFN-γ production. We found that IL-12 or IL-18 alone could not upregulate miR-146a; although, the former can induce a moderate level of IFN-γ production. These data imply that moderate stimulation would not initiate the upregulation of miR-146a to have a stronger inhibition of IFN-γ production; only when NK cells receive excessive co-stimulation of IL-12 and IL-18 or become super active, the effect from the miR-146a induction would be launched to tune the function of NK cells into an appropriate range. Of note, the combination of IL-12 and IL-18 is actually one of the most potent stimuli, if not the most potent one, for IFN-γ production by NK cells.

Two functionally distinct subsets of human NK cells can be defined by the intensity of CD56 surface expression (37). CD56^dim^ cells are generally considered more cytotoxic, whereas CD56^bright^ cells are highly responsive to cytokines to have maximum IFN-γ production (33, 38). Our data showed that the resting CD56^bright^ NK cell subset exhibited significantly higher miR-146a expression compared to the CD56^dim^ NK cell subset. Simultaneously, the resting CD56^bright^ NK cell subset constitutively possessed relatively higher levels of NF-κB activity than that of the resting CD56^dim^ NK cell subset. NF-κB activation in the resting CD56^bright^ NK cell subset could regulate IFN-γ production positively and probably induced transcription of pri-miR-146a, leading to increased mature miR-146a that could prevent NK cells from overproducing IFN-γ by inhibiting NF-κB activation. It will be very important to prevent CD56^bright^ NK cell subset from overproducing IFN-γ, suggesting a possible explanation for the elevated levels of miR-146a in CD56^bright^ NK cell subset. This further substantiates our hypothesis that the effect from excessive miR-146a may only occur in NK cells with excessive IFN-γ production or with that capacity.

Some previous studies have suggested that miR-146a plays roles in regulating both the innate and adaptive immune response (25, 39–41). A previous study showed that miR-146a levels increased following LPS exposure and negatively correlate with the level of TNF-α when monocytes develop a state of LPS tolerance. Transfection of miR-146a is sufficient to induce endotoxin tolerance, a hyporesponsive state of monocytes, even in the absence of LPS-priming (42). Although the inflammatory response is important for pathogen clearance, it takes a toll on the body, which can cause a serious disease if left unregulated. It has been reported that low levels of miR-146a in lupus patients correlate with higher levels of interferon and with worse symptoms (43). miR-146a has been also known to play an important role in regulating the adaptive immune response. The expression of miR-146a has been shown higher in Th1 cells and shown lower in Th2 cells when compared to naïve T cells (44). Recently, Lu and colleagues showed that miR-146a expression is enriched in regulatory T (Treg) cells and it is critical for Treg suppressor function *in vivo*. Knock-out of miR-146a expression in the Treg cells of mice results in an increase in the percentage of IFN-γ-producing T-cell subset and a fatal break down of tolerance, resulting in a CD4^+^ helper T lymphocyte (Th1)-mediated immunopathology, which is mediated by IFN-γ (45). In addition, miR-146a may promote the survival of self-reactive T cells in autoimmunity, as miR-146a levels in both synovial tissues and PBMCs of rheumatoid arthritis patients are increased (46, 47). Together, these studies depict a key role for miR-146a as a negative regulator of pro-inflammatory signaling in both innate and adaptive immunity and miR-146a may play a role in autoimmune diseases. Here, we focused on NK cells and found that miR-146a negatively regulated IFN-γ production, which is supported by a recent study reported in the setting of chronic hepatitis B and hepatic carcinoma (48). As aforementioned, our mechanistic characterization supports a unique restriction role of miR-146a in NK cells highly expressing IFN-γ.

There are multiple signaling pathways to control IFN-γ gene expression and its production, including positive signaling pathways, such as the MAPK signaling pathway, the JAK–STAT signaling pathway, the T-BET signaling pathway, and the NF-κB signaling pathway, as well as negative regulation *via* the TGF-β signaling pathway (49). Many of the synergistic stimuli that enhance IL-12-mediated IFN-γ production by NK cells share the ability to activate the transcription factor NF-κB (28). Recent studies have confirmed that miR-146a is a transcriptional target of NF-κB; it is hypothesized that miR-146a may serve as a feedback inhibitor of NF-κB activation (25, 50). In our study, we have provided functional evidence that miR-146a negatively regulates the NF-κB signaling pathway and subsequently results in less IFN-γ in human NK cells. How to integrate miR-146a and its associated NF-κB signaling pathway with other signaling pathways regulating IFN-γ gene expression requires further studies.

Our work suggests an addition of miR-146a to the list of negative regulators of NK cell IFN-γ production. Although our current study focuses on the negative role of miR-146a in regulating IFN-γ production, a recent study suggests that miR-146a may also negatively regulate cytotoxicity of NK cells (48). NK cells function in immune surveillance to eradicate both virus-infected cells and tumor cells. Whether viral infection, as it has been shown in tumor cells (48), can upregulate the expression of miR-146a in NK cells, reduce the secretion of IFN-γ, and thus inhibit the immune clearance, resulting in virus invasion, is also warranted for further studies.

In brief, the present study suggests that miR-146a downregulates NF-κB signaling at least *via* targeting IRAK1 and TRAF6 and functions as a novel negative regulator of NK cell IFN-γ production that helps to fine-tune the immune response of NK cells. Our findings strengthen an approach to prevent or treat virus infections, autoimmune diseases, and cancer by targeting miR-146a and its involved signaling pathways.

## Ethics Statement

This study was carried out in accordance with the institutional review board of The Ohio State University. The protocol was approved by the institutional review board of The Ohio State University.

## Author Contributions

Conception and design: JY. Development of methodology: HW, YZ, XW, YW, HC, XL, and NT. Analysis and interpretation of data (e.g., statistical analysis, biostatistics, computational analysis): HW and JZ. Writing and/or revision of the manuscript: HW, JY, and YW. Administrative, technical, or material support (i.e., reporting or organizing data, constructing databases): HW and JY. Study supervision: JY and YP.

## Conflict of Interest Statement

The authors declare that the research was conducted in the absence of any commercial or financial relationships that could be construed as a potential conflict of interest. The reviewer HH and handling editor declared their shared affiliation.
