# Supplementary material for: Regulation of Human Natural Killer Cell IFN-γ Production by MicroRNA-146a via Targeting the NF-κB Signaling Pathway
Source: Front Immunol. 2018 Mar 9;9:293. doi: 10.3389/fimmu.2018.00293 (PMC5854688; doi:10.3389/fimmu.2018.00293)
Supplement: Supplementary file 1 [file presentation_1.pdf]

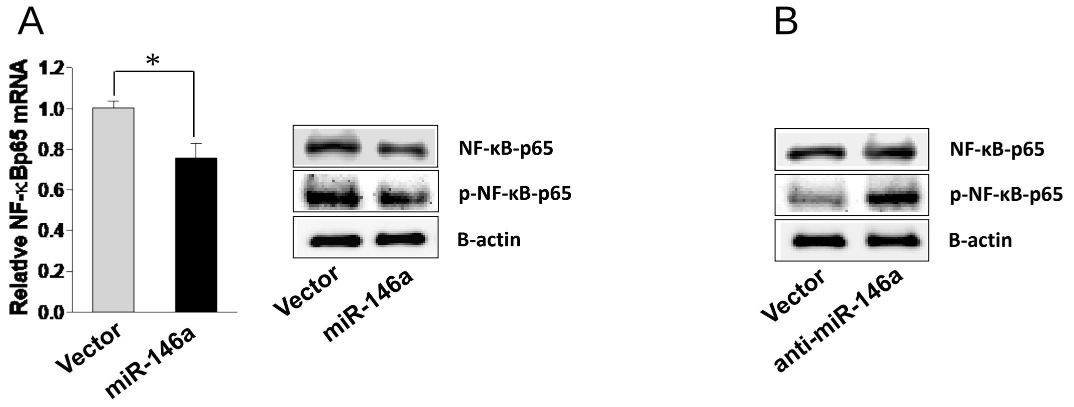

Figure S1 NF-κB signaling is negatively regulated by miR-146a in resting and cytokine-stimulated human NK cells.

NK-92 cells expressing miR-146a or anti-mIR-146a were cultured in medium without IL-2 for 24 h. Cells were harvested to extract RNA, followed by cDNA synthesis. Then the expression of NF-κB p65 at the mRNA level was quantified by qRT-PCR (A). NF-κB p65 protein levels and its activity were measured by immunoblotting (A, B). The reported results are representative of at least three donors with similar results. Data shown are mean±SD. *, p<0.05; **, p<0.01. Error bars represent SD.
